# Self-prioritization with unisensory and multisensory stimuli in a matching task

**DOI:** 10.3758/s13414-022-02498-z

**Published:** 2022-05-10

**Authors:** Clea Desebrock, Charles Spence, Ayla Barutchu

**Affiliations:** Department of Experimental Psychology, Crossmodal Research Laboratory, University of Oxford, Oxford, OX2 6GG UK

**Keywords:** Self-prioritization, Matching, Simple detection, Multisensory, Self-relevance, Auditory labels, Blocked vs. intermixed

## Abstract

A shape-label matching task is commonly used to examine the self-advantage in motor reaction-time responses (the Self-Prioritization Effect; SPE). In the present study, auditory labels were introduced, and, for the first time, responses to unisensory auditory, unisensory visual, and multisensory object-label stimuli were compared across block-type (i.e., trials blocked by sensory modality type, and intermixed trials of unisensory and multisensory stimuli). Auditory stimulus intensity was presented at either 50 dB (Group 1) or 70 dB (Group 2). The participants in Group 2 also completed a multisensory detection task, making simple speeded motor responses to the shape and sound stimuli and their multisensory combinations. In the matching task, the SPE was diminished in intermixed trials, and in responses to the unisensory auditory stimuli as compared with the multisensory (visual shape+auditory label) stimuli. In contrast, the SPE did not differ in responses to the unisensory visual and multisensory (auditory object+visual label) stimuli. The matching task was associated with multisensory ‘costs’ rather than gains, but response times to self- versus stranger-associated stimuli were differentially affected by the type of multisensory stimulus (auditory object+visual label or visual shape+auditory label). The SPE was thus modulated both by block-type and the combination of object and label stimulus modalities. There was no SPE in the detection task. Taken together, these findings suggest that the SPE with unisensory and multisensory stimuli is modulated by both stimulus- and task-related parameters within the matching task. The SPE does not transfer to a significant motor speed gain when the self-associations are not task-relevant.

**Supplementary Information:**

The online version contains supplementary material available at 10.3758/s13414-022-02498-z.

## Introduction

Self-representation is widely held to guide our cognition and action, and self-relevance has repeatedly been shown to influence stimulus processing (Cunningham & Turk, 2017; Sui & Humphreys, 2017b). Using stimuli such as our own face or name, our attributes (e.g., personality traits), our property, and also those stimuli that are newly and arbitrarily associated with the self, studies have investigated self-relevance across a diverse range of tasks. Self-related stimuli have been shown to modulate attention (Alexopoulos et al., 2012; Brédart et al., 2006; Humphreys & Sui, 2016; Moray, 1959), perceptual decision-making (Constable et al., 2019; Golubickis et al., 2017; Humphreys & Sui, 2016), memory (Rogers et al., 1977; Symons & Johnson, 1997; Yin et al., 2019), and overt movements (Constable et al., 2011; Constable et al., 2014; Desebrock et al., 2018; Desebrock & Spence, 2021).

A paradigm for investigating the effects of self-relevance without the confounds of stimulus familiarity (inherent in own-name or -face stimuli, for example) was established with the introduction of Sui et al.’s (2012) matching procedure. In an initial learning phase, participants are instructed to associate geometric shapes with visually presented labels referring to people (e.g., self-circle, friend-triangle, stranger-square). Arbitrary associations between the identities and the stimuli are thus formed rapidly. In the main task, participants then indicate whether shape-label stimulus pairs match or mismatch the newly learned associations, typically by means of keypress responses. Reaction times (RTs) to the self-associated shape-label stimulus pairs are consistently found to be shorter and more accurately selected. This phenomenon is known as the Self-Prioritization Effect (SPE; Sui et al., 2012). Self-prioritization can thus be measured without the need to use stimulus objects that are highly familiar. Self-prioritization appears to be dissociable (at least in part) from the effects of stimulus familiarity, emotional valence, and reward (Schäfer et al., 2020a; Stolte et al., 2017; Stolte et al., 2021; Sui et al., 2012; Woźniak & Knoblich, 2019; Yankouskaya et al., 2017). Furthermore, distinct neural circuitry underpins the SPE (Sui et al., 2013; Yankouskaya et al., 2017).

## The effect of sensory modality on the Self-Prioritization Effect (SPE)

Typically, the object and label stimuli used in the matching task have been visual (e.g., Golubickis et al., 2017; Sui et al., 2012; Woźniak et al., 2018), and the SPE has been interpreted as arising from the binding of the visual self-associated stimulus to the self-concept (Humphreys & Sui, 2015; Schäfer et al., 2020a). However, it has previously been assumed (Humphreys & Sui, 2016) and later evidenced, in studies using auditory and tactile objects, that the SPE in the matching task is not visual-specific (Payne et al., 2020; Schäfer et al., 2015; Schäfer et al., 2016b; Stolte et al., 2021). Furthermore, it has been suggested that the self-representation underpinning self-prioritization may be modality-general (or ‘abstract’; Woźniak et al., 2018). Indeed, Schäfer et al. (2016b) documented that the magnitude of the SPE did not appear to differ in responses to visual as compared with auditory and tactile object stimuli (with objects presented 500 ms before the visual label stimuli), suggesting that the SPE was underpinned by neural processes that are common to responses across all sensory stimuli. The authors concluded that the SPE was therefore likely to be a modality-general mechanism.

In contrast, Stolte et al. (2021) used simultaneous presentations of auditory objects and visual labels (as such creating the opportunity for multisensory integration) and documented that the SPE was smaller than to the standard visual shape and visual label stimulus pairs. The authors suggested that under some conditions vision may dominate for self-associations (Stolte et al., 2021). They also found that the SPE and visual dominance can be modulated by the relative frequencies of the auditory tones used to pair with the self-associated labels. As Hutmacher (2019) points out, visual dominance is socially and culturally reinforced. We tend to rely more on visual than auditory (or other sensory) information (Sinnett et al., 2007), and actively attend to visual over auditory stimuli in certain task contexts (Posner et al., 1976). Any task-induced visual dominance may be expected to reduce the SPE with auditory stimuli.

## Task design, stimulus parameters, and the SPE

Perhaps unsurprisingly, certain stimulus parameters and elements of the task design differed across the previous studies that have compared the SPE in responses to both visual and auditory stimuli (Schäfer et al., 2016b; Stolte et al., 2021). For example, Schäfer et al. presented their auditory stimuli at 50 dB, and used a blocked-trial design (with responses to auditory and visual stimuli being assessed in separate experiments). These authors documented that the magnitude of the SPE did not differ in responses to visual as compared to auditory stimuli. By contrast, Stolte et al. presented their auditory stimuli at 75 dB, and used an intermixed presentation with all stimulus modality types randomly intermixed within a block of experimental trials. These authors documented that the SPE in responses to paired auditory object and visual label stimuli was smaller than to the standard visual shape and visual label stimulus pairs. Such differences in the stimulus parameters and task design across studies may differentially influence the SPE across visual and auditory stimuli.

### Blocked versus intermixed trials

Task design (i.e., whether trials are intermixed or blocked by the identity of the target shape) has previously been demonstrated to influence self-prioritization. In a recent study using visual stimuli in the matching task, the authors documented that the SPE was greater in intermixed trials (using self- and friend-associated shapes) than in blocked trials (i.e., using self- or friend-associated shapes; Golubickis & Macrae, 2021). Blocking or intermixing trials by stimulus modality may also modulate the SPE. In intermixed as compared with blocked trials, responding to the randomised unisensory and multisensory trials requires modality-switching. Modality-switching typically results in slower and less accurate responses (Spence et al., 2001), reflecting ‘switching costs’ (Barutchu & Spence, 2021; Otto & Mamassian, 2012). Sensory switch costs often result in slower responses to unisensory stimuli since attention must be shifted between the senses (Kreutzfeldt et al., 2015; Lukas et al., 2010; Spence et al., 2001). In contrast, responses to multisensory stimuli require no switching since processing just one of the two sensory stimuli is sufficient to produce a valid motor response in those detection paradigms with redundant signals. Switching can thus inflate multisensory gains in detection paradigms (Barutchu & Spence, 2021; Otto & Mamassian, 2012; Shaw et al., 2020). In a matching task, on the other hand, both signals need to be processed in order to make a decision and thereafter a valid motor response; therefore a switch to a multisensory signal in a matching task may be expected to lead to a multisensory cost. Indeed, multisensory costs were observed in Stolte et al.’s (2021) study, along with a reduced SPE in responses to those audiovisual stimuli. Switching could thus (partly) underpin the reduced SPE with audiovisual stimuli in their study, which was further investigated in the present study.

Increased demands on working memory in intermixed trials, as compared with blocked trials, may also favour those responses involving the stronger of the object-label associations (Golubickis & Macrae, 2021); in other words, those involving visual self-associations (should they be stronger than their auditory counterparts). Certain self-related categories appear to be prioritized more than others when placed into competition (Enock et al., 2020; Turner et al., 1987); for example, in intermixed trials (Enock et al., 2020). Automatic self-prioritization has also been demonstrated in endogenous attentional processes in working memory tasks (Yin et al., 2019). Self-prioritization with auditory stimuli may thus be diminished when auditory or audiovisual self-associations compete with (putatively stronger) visual self-associations in intermixed trials.

Alternatively, visual dominance arising in intermixed trials as a result of attentional effects could reduce the SPE with auditory stimuli. For example, the alerting properties of auditory stimuli are thought to elicit an override response in participants such that they actively focus their attention toward the weaker visual stimuli as a consequence. Fewer cognitive resources thus remain to attend to, and process, auditory stimuli (Posner et al., 1976). Stolte et al. (2021) examined the SPE across visual and audiovisual stimuli only in the matching task, so it is not known whether the SPE would be similarly reduced with unisensory auditory stimuli in the task (i.e., auditory-only object and label stimuli).

### Auditory stimulus intensity

The SPE has been shown to be affected by low-level sensory features of visual stimuli (i.e., stimulus contrast): namely, self-bias increased when stimulus contrast was reduced (Sui et al., 2012 – Experiment 4). Sui et al. (2012) suggested that this experimental manipulation could be considered a perceptual modulation. As noted, in contrast to Stolte et al. (2021), Schäfer et al. (2016b) presented their auditory stimuli at 50 dB rather than 75dB. Here it is worth bearing in mind that sound intensity has been shown to modulate modality-specific (perceptual) processes, as well as the motor output (Miller et al., 1999; St Germain et al., 2020), and also multisensory processes (Barutchu et al., 2010; Ma et al., 2009). If the SPE were to interact with stimulus intensity, it might be expected to increase with auditory stimulation at lower as compared with higher intensities. In other words, if self-relevance in the matching task can boost auditory perceptual processes as has been proposed for visual perceptual processes, the SPE would increase if reduced auditory stimulus intensity is detrimental to stranger-associated, but not self-associated, responding.

### Modalities of the stimulus objects and labels

Typically, previous studies examining the SPE with auditory and visual stimuli have used visual labels (Payne et al., 2020; Schäfer et al., 2015; Schäfer et al., 2016b; Stolte et al., 2021). To date, no studies have examined the SPE with unisensory auditory stimuli (simultaneously presented auditory object and auditory label). That said, the self-bias arising with unisensory auditory stimuli (e.g., the participant’s own name) has been demonstrated in other paradigms, for example, in dichotic listening tasks (e.g., Moray, 1959). Notably, however, the auditory self-bias that was first shown in Moray’s early research was exhibited by only ≈33% of the participants (Conway et al., 2001; Wood & Cowan, 1995). Furthermore, differential mechanisms (with which the SPE could potentially interact) have been shown to underlie the processing of visual and auditory linguistic and object stimuli carrying seemingly equivalent information (Arana et al., 2020; Chen & Spence, 2018). The sensory modality of the label (auditory or visual) may therefore not be interchangeable, and the SPE may be moderated by the combination of object/label modality type. It could be that visual object-label self-associations are more easily formed, or accessed, than unisensory auditory or multisensory object-label self-associations. Alternatively, however, *unisensory* (rather than just visual) self-associations may be more easily formed or accessed than multisensory self-associations.

Attention has also been shown to modulate the SPE (Humphreys & Sui, 2016), and attentional effects arising in responses to auditory and audiovisual stimuli may also interact with the SPE. For example, while attentional-capture is likely to increase with higher-intensity sounds, when those sounds are presented simultaneously with visual stimuli (i.e., as a multisensory warning signal), such effects may be attenuated (Spence & Driver, 1999). In other words, the SPE may be moderated across unisensory auditory and audiovisual stimuli.

## The present study

The present study explored whether stimulus-related and task-design parameters would moderate the SPE in a matching task requiring motor responses to unisensory and multisensory stimuli. We also examined (for the first time) whether the SPE would transfer to a multisensory simple detection motor response paradigm whereby, in contrast to the matching task, the associations of the stimuli with the self were irrelevant to the task at hand (cf. Orellana-Corrales et al., 2021; Stein et al., 2016; Wade & Vickery, 2018; Woźniak & Knoblich, 2021).

**Fig. 1:**
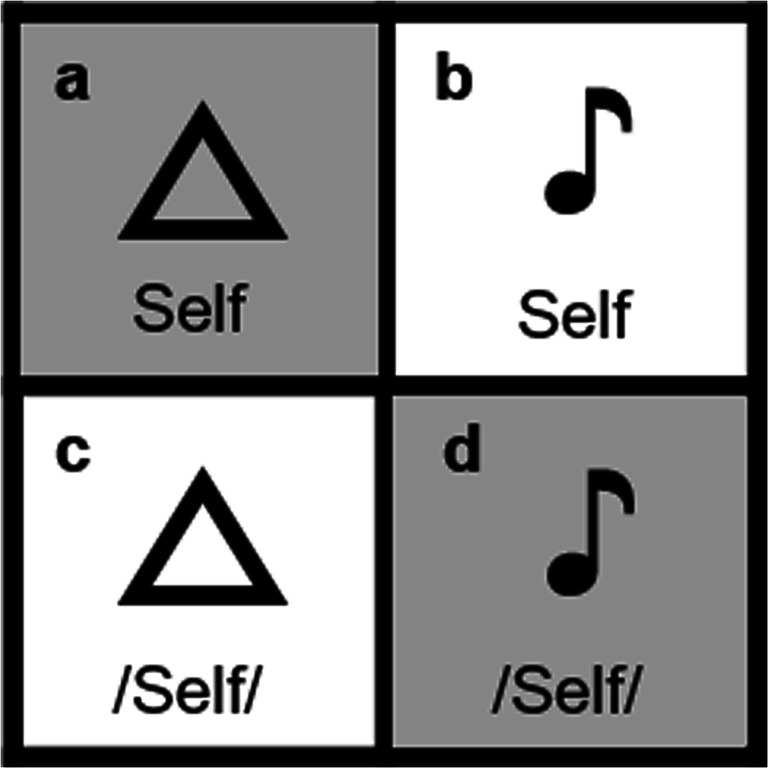
Unisensory stimuli and multisensory stimuli used in the matching task. Example using the self-associated label. Simultaneously-presented: **a.** Unisensory visual stimulus type: Visual object + Visual Label = V+VL. **b.** Multisensory stimulus type: Auditory Object + Visual Label = A+VL. **c.** Multisensory stimulus type: Visual object + Auditory label = V+AL. **d.** Unisensory auditory stimulus type: Auditory object + Auditory label = A+AL. ♪ = categorical sound. **△** = geometric shape (triangle presented as example). The Self label is depicted. /Self/ denotes the spoken self-associated label

In order to compare the SPE in the matching task across unisensory auditory stimuli, as well as unisensory visual and multisensory stimuli, we introduced auditory labels (see Fig. 1). The use of auditory labels also ensured equal probability of occurrence of the visual and auditory stimuli across all trials. (In a version of the matching task using visual and auditory object stimuli, with only visual label stimuli, a visual stimulus but not an auditory stimulus would be presented in every trial).

Two independent groups of participants took part in the present study to compare the SPE across auditory stimulus intensities. The participants in Group 1 completed an audiovisual adaptation of the matching task (Sui et al., 2012), responding to unisensory auditory, unisensory visual, and multisensory object-label pairs, in both blocked and intermixed trials, and with the auditory stimuli presented at 50 dB (following Schäfer et al., 2016b). For the participants in Group 2, the procedure was identical except that the auditory stimuli in the matching task were presented at 70 dB (consistent with Stolte et al., 2021). The participants in this latter group then subsequently also completed a simple detection task in which they made motor responses to the shape and sound stimuli and their multisensory combinations. Previous studies have examined whether the self-associations formed in the matching task can transfer to other task paradigms (Chiarella et al., 2020; Dalmaso et al., 2019; Hu et al., 2020; Moradi et al., 2018; Payne et al., 2017; Stein et al., 2016; Woźniak & Knoblich, 2019; Yin et al., 2019). To the best of our knowledge, though, the present study is the first to examine whether the SPE can transfer across tasks to a simple multisensory detection motor response paradigm.

It was expected that the SPE in the matching task would be moderated by the block type (blocked or intermixed trials), and reduced with audiovisual as compared to visual stimuli (e.g., Stolte et al., 2021), although the latter may depend on task context (block type and auditory stimulus intensity; cf. Schäfer et al., 2016b). It was further hypothesized that while a modulation of the SPE across stimulus modality types might be expected (i.e., if the SPE interacts with modality-specific processes or differential stimulus effectiveness), a consistently reduced SPE with auditory stimuli would lend support to Stolte et al.’s findings. An equivalent SPE across stimulus modality types, on the other hand, would lend support to the contention that the SPE is underpinned by a modulation of modality-general processes (in line with Schäfer et al., 2016b). In addition, if an SPE arose in the detection task, this would further suggest that the unisensory and multisensory self-associations established in the matching task can be automatically activated in fast motor responses to the unisensory and multisensory stimuli.

## Methods

### Participants

#### Matching tasks

In the present study, the SPE was examined using a three-way 2 × 4 × 2 mixed design: 2 between groups (Group 1 in which the auditory stimuli were presented at 50 dB, and Group 2 in which the auditory stimuli were presented at 70 dB), and, within each group, we examined the effects of the four sensory modality stimulus types (unisensory visual, unisensory auditory, multisensory with visual labels, multisensory with auditory labels), × 2 block types (trials blocked by sensory modality type, intermixed trials of the unisensory and multisensory stimuli). Multisensory gains in the matching task were examined using a 2 (Auditory stimulus intensity between-groups Groups 1 and 2: 50 dB, 70 dB) × 2 (within-groups Block type: blocked, intermixed) × 2 (within-groups AV Stimulus type: A+VL, V+AL) × 2 (within-groups Association: self, stranger) mixed factorial structure. Previous studies reported a large self-bias effect using the standard visual modality matching task (*dz* > 0.80; Sui et al., 2012) and a medium effect-size using an adaptation of the task using auditory objects and visual labels in a blocked-trial design (*dz* = 0.51; Schäfer et al., 2016b). Stolte et al. (2021) reported a large effect size for the interaction between association and stimulus type using an intermixed-trial design (η^2^ = .15; Stolte et al., 2021 – Table 1). For a medium effect size (η_p_^2^ = .12), a probability of 1−β=0.80, and an α-value of 0.05, a minimum sample size of 28 participants was required for a 2 × 4 × 2 between-within-within ANOVA and 60 for a 2 × 2 × 2 × 2 between-within-within-within ANOVA (MorePower 6.0.4 program; Campbell & Thompson, 2012

**Table 1:** Mean RT, Accuracy (percentage correct), and Sensitivity (D-Prime; *d*′) index scores with Standard Deviations, as a Function of Stimulus Type (Visual-shape+Visual-Label, A+AL, A+VL, V+AL), Association (self, stranger), Block Type (blocked, intermixed), and Match Condition (match, mismatch) in the Matching tasks

Stim. type	Assoc.	RT				Percentage correct				*d*′	
		Blocked		Intermixed		Blocked		Intermixed		Block	Intermixed
		Match	Mismatch	Match	Mismatch	Match	Mismatch	Match	Mismatch		
Group 1 (50dB) (*N* = 28)											
V+VL	Self	674 (57)	752 (68)	720 (63)	808 (62)	92 (9)	81 (14)	86 (10)	82 (13)	2.65 (0.93)	2.27 (0.87)
	Stranger	735 (76)	763 (62)	771 (71)	791 (57)	80 (16)	84 (11)	81 (14)	83 (11)	2.20 (1.05)	2.06 (0.95)
A+AL	Self	714 (88)	791 (69)	805 (60)	862 (48)	86 (12)	77 (18)	79 (17)	71 (17)	2.12 (1.11)	1.55 (1.02)
	Stranger	757 (64)	764 (56)	853 (61)	844 (53)	79 (17)	83 (15)	63 (17)	74 (18)	2.02 (1.05)	1.12 (1.06)
A+VL	Self	673 (88)	744 (59)	776 (63)	835 (60)	91 (11)	87 (12)	85 (16)	77 (19)	2.87 (1.08)	2.08 (1.19)
	Stranger	732 (71)	721 (56)	812 (71)	814 (57)	86 (11)	89 (9)	75 (16)	78 (17)	2.69 (0.89)	1.71 (1.06)
V+AL	Self	627 (83)	717 (55)	719 (57)	790 (56)	92 (9)	90 (10)	87 (10)	86 (11)	3.00 (0.83)	2.49 (0.83)
	Stranger	720 (78)	715 (58)	780 (70)	772 (53)	86 (10)	93 (6)	81 (14)	87 (11)	2.91 (0.97)	2.20 (0.89)
Group 2 (70dB) (*N* = 22)											
V+VL	Self	664 (75)	769 (71)	700 (46)	784 (56)	90 (15)	80 (11)	87(10)	80 (19)	2.48 (0.94)	2.22 (1.00)
	Stranger	716 (82)	758 (64)	745 (68)	775 (54)	80 (17)	85 (14)	83 (15)	82 (15)	2.38 (1.35)	2.23 (1.21)
A+AL	Self	714 (79)	794 (55)	809 (84)	858 (73)	87 (10)	81 (17)	73 (17)	65 (20)	2.38 (1.24)	1.18 (1.03)
	Stranger	746 (105)	769 (71)	811 (96)	841 (67)	78 (20)	82 (16)	65 (23)	70 (22)	2.06 (1.34)	1.15 (1.34)
A+VL	Self	683 (71)	781 (78)	777 (68)	836 (71)	93 (7)	84 (14)	86 (10)	72 (17)	2.76 (1.09)	1.84 (0.84)
	Stranger	735 (96)	746 (65)	801 (94)	819 (64)	82 (13)	89 (9)	73 (19)	80 (15)	2.41 (0.84)	1.72 (1.09)
V+AL	Self	628 (79)	726 (97)	713 (64)	771 (73)	95 (5)	86 (15)	89 (9)	84 (19)	3.05 (0.99)	2.58 (1.05)
	Stranger	696 (111)	711 (101)	744 (73)	763 (75)	87 (12)	90 (9)	82 (16)	88 (16)	2.77 (1.09)	2.51 (1.28)

Note. *RT* Reaction time, Standard deviations appear within parentheses. *Stim. type* Stimulus type, *Assoc*. Association

Sixty right-handed participants (14 male, ages 18–23 years, mean age 18.92 ± 1.00) with self-reported normal or corrected-to-normal visual acuity and hearing took part in the study. In Group 1, there were 31 right-handed participants (seven male, ages 18–23 years, mean age 19.03 ± 1.02); in Group 2, there were 29 right-handed participants (seven male, ages 18–22 years, mean age 18.79 ± 0.98). The participants were recruited via the Oxford University Research Participation Scheme and received course credit for their time and effort. A written consent form approved by the University of Oxford Central University Research Ethics Committee (MS-IDREC- R61057/RE002) was completed by all participants.

#### Detection task

No previous studies have looked at the SPE in a multisensory simple detection task, and so the effect size was unknown. Given that the effect size of the SPE using visual or auditory stimuli is typically medium to large (Schäfer et al., 2016b; Sui et al., 2012), a moderate effect size (*f* = 0.25), a probability of 1−β=0.80, and an α-value of 0.05, would require a minimum sample size of 16 participants. (G*Power 3.1 program; Faul et al., 2009). In Group 2, 29 right-handed participants (seven male, ages 18–22 years, mean age 18.79 ± 0.98) with normal or corrected-to-normal visual acuity and hearing completed the matching task. Twenty-six participants (seven male, ages 18–22 years, mean age 18.73 ± 1.00) of the 29 who completed the matching task completed the detection task (three participants who completed the matching task did not complete the detection task).

### Stimuli and apparatus

#### Matching tasks

All computer tasks were conducted on a PC with a 23-in. LCD monitor (1,920 × 1,080 pixels at 60-Hz refresh rate) using E-Prime software (version 2.0). A QWERTY-keyboard recorded button-press responses. Following previous studies (Sui et al., 2012), participants made matching and mismatching responses using their index and middle finger of their right hand on two adjacent keyboard keys. Visual object stimuli consisted of two geometric shapes (V) from the following set (pentagon, hexagon, or octagon, each subtending 3.2 × 3.2° of visual angle), and two written-text self- and stranger-related labels (VL) (*your*, *their*, referring to ‘your shape’, ‘their shape’), subtending a visual angle of 2.1 × 0.7°. The words ‘Your’ and ‘Their’ were chosen as labels due to their similar word length and equivalent number of syllables (durations), and equivalent low ratings for ‘word concreteness’ (Brysbaert et al., 2014; see Desebrock & Spence, 2021). The two shapes allocated to each participant and the labels that they were paired with were counterbalanced across participants following a Latin square design. The same shape and label pairs were maintained for the blocked and intermixed presentations. The shapes and labels were presented against a black background in the centre of the PC-screen. The shape was positioned above (and the label below) a fixation cross (0.6 × 0.6° of visual angle).

Auditory object stimuli (A) consisted of two neutral instrumental sound samples (150-ms duration) from the following set (violin, synth vocal, and clarinet). The neutral instrumental sound samples were selected from two validated, publicly available sound sets: the Musical Emotional Bursts (MEB; Paquette et al., 2013) and the Montreal Affective Voices (MAV; Belin et al., 2008). The MEB is a set of musical affect bursts expressing basic emotional states (specifically, happiness, sadness, and fear) and also ‘neutral’ expressions. Two neutral sounds from the MEB and one neutral sound sample from the MAV on the same musical note were selected for the present study: “V3_NEUTRAL_MEB” (violin), “45_NEUTRAL_MAV” (vocal), and “C1_NEUTRAL_MEB” (clarinet). All sound samples were trimmed to a duration of 150 ms. The auditory labels (AL) were two recorded samples (bursts) of fast natural speech utterances of the words “your” and “their” spoken by a female actor. The auditory stimuli were presented via two freestanding loudspeakers, one placed on either side of the PC screen at a distance of 50 cm from the participant. Schäfer et al. (2016b) used auditory stimuli of low intensity (50 dB) just above ambient sound levels in their sound and visual label version of the matching task. In the present study the sound pressure level (SPL) of the ambient background noise was set at 45 dB. The auditory stimuli were presented at 50 dB SPL (following Schäfer et al., 2016b) to one group of participants (Group 1; N = 31) and at 70 dB (consistent with Stolte et al., 2021) for a second group (Group 2; N = 29). Signal-to-noise (SNR) ratios were thus 5 dB and 25 dB for the two groups, respectively. Auditory and visual object-label stimuli were combined to produce four stimulus types: visual only (object and label both visual = V+VL), auditory only (sound and label both auditory = A+AL), auditory with visual label (A+VL), and visual object with auditory label (V+AL). All of the stimuli were presented simultaneously for 150 ms.

#### Detection task

A Cedrus RB-530 response-box recorded button-presses in the simple detection task. (A response box was used for this task due to the robust nature, size, and spacing of the response-box buttons which could accommodate the fast motor responses required by the detection task – the response box was only used for the simple detection task.) The stimuli consisted of the two self- and stranger-associated geometric shapes and instrumental sounds that the participant had been assigned in the preceding matching tasks. (NB: The visual and auditory labels used in the matching task were not used as stimuli in the detection task.)

### Procedure

#### Matching tasks

Participants carried out an extended multisensory version of Sui et al.’s (2012) computer-based matching task, in a single experimental testing session. The duration of the testing session ranged from 90 to 120 min. The matching task consisted of four blocks of blocked trials (one stimulus type per block; 80 trials per block), the order of which was counterbalanced across participants following a balanced Latin square design, followed by five blocks of randomly intermixed trials (all four stimulus types presented randomly with equal probability within each block; 96 trials per block). Familiarity with both the matching task and the 16 stimulus-response mappings (2 × Association × 2 Match-type × 4 stimulus types) gained during the blocked trials enabled participants to complete the intermixed trials, which were otherwise too difficult. (See Appendix 6 for further details regarding task order.)

Following Sui et al.’s (2012) procedure, for the visual stimulus blocks (V+VL), participants were instructed (via onscreen text) to associate one of their allocated geometric shapes or sound with themselves (specifically, as ‘your’ shape; e.g., ‘the pentagon is your shape’) and the second allocated shape or sound with ‘a stranger’ (as ‘theirs’; e.g., ‘the octagon is their shape’). Order of instructions pertaining to ‘self’ and ‘stranger’ associations were counterbalanced across participants. Following this, the participants carried out the matching task in which participants used their right hand to depress one of two response keys (b or v) on the keyboard to indicate either a ‘matching’ or a ‘mismatching’ judgment (the keys and match/mismatch mappings were counterbalanced across participants). The participants were instructed to make their responses to the stimuli as rapidly and accurately as possible. The main task was preceded by 24 practice trials, with a performance accuracy threshold set at 60% correct (that is, participants had to achieve at least 60% correct before they could proceed to the main task). Onscreen feedback was presented (*Correct*, *Incorrect*, *Too Slow*) during the practice trials, with the ‘*Correct*’ feedback omitted during the main task (following Schäfer et al., 2016b).

**Fig. 2:**
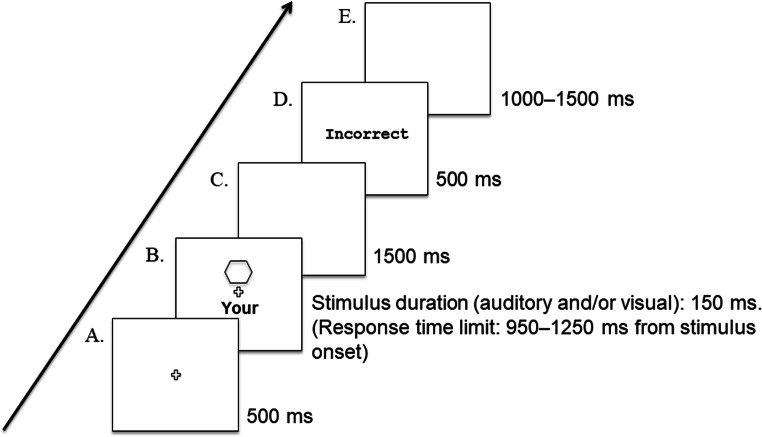
Schematic overview of an experimental trial sequence for the matching task. (Displayed elements not to scale). **a** Fixation cross. **b** Visual, auditory, or audiovisual stimulus onset. Shape-label (*Visual Task*) stimulus shown. **c** Blank screen. **d** Written feedback displayed on screen – “Incorrect” / “Too slow” (for a ‘correct’ response a blank slide was displayed following Schäfer et al., 2016b). E. Inter-trial intervals generated at random

The procedure was the same for all blocks except that in the A+VL block, participants matched instrumental sounds with visual labels (e.g., clarinet-‘your’, violin-‘their’), in the V+AL block they matched shapes with auditory labels, in the A+AL block they matched the instrumental sounds with the auditory labels. In intermixed blocks, the participants matched the visual and auditory labels with the shapes and sounds that they had been allocated in the previous blocks, with all stimulus modality types intermixed within each block of trials. Prior to carrying out the intermixed-trial blocks, the participants completed 32 practice trials. There was an 8-s break between each block of trials. There were 800 trials in total (320 blocked trials, and 480 intermixed trials), across 32 conditions – 2 block types (blocked/intermixed) × 4 stimulus types (V+VL, A+AL, A+VL, V+AL) × 2 associations (self/stranger) × 2 matching types (match/mismatch), 20 trials per condition in blocked trials, and 30 trials per condition in the intermixed trials. Each condition was randomly generated with an equal number of presentations. The participants were informed of their overall accuracy at the end of each block of trials. A schematic representation of an experimental trial in the matching task is shown in Fig. 2

#### Detection task

After completing the matching task, the participants in Group 2 completed a simple detection task in which they were asked to respond as rapidly and accurately as possible to the auditory (instrumental sounds), visual (shapes), and audio-visual (shape-sound) stimuli used in the matching task. In a modified version of the typical detection task, a short response time limit of 300 ms was used to ensure that participants did not delay their responses (to avoid the intentional evaluation of the stimuli), and to keep participants alert to the task, responding as rapidly as they could (< 3% of reaction times (RTs) were excluded based on this response limitation). Only the shape and sound stimuli were used (the labels were not used). As such, the stimuli pairs in the detection task differed from those in the matching tasks (which used labels with individual shape/sound stimuli). The unisensory auditory (self- or stranger-associated), unisensory visual (self- or stranger-associated), and audio-visual stimuli (self-match, stranger-match, stranger-mismatch, and self-mismatch) were presented in a random order across four blocks of 80 trials. Self-match trials consisted of the self-associated shape and self-associated sound stimuli, and stranger-match trials consisted of the stranger-associated shape and stranger-associated sound stimuli. The stranger mismatch trials consisted of the stranger-associated shape and self-associated sound stimuli, while the self-mismatch trials consisted of the self-associated shape and stranger-associated sound stimuli. There was a total of eight conditions: A self-associated unisensory auditory stimulus type, a stranger-associated unisensory auditory stimulus type, a self-associated unisensory visual stimulus type, a stranger-associated unisensory visual stimulus type, two self-associated audiovisual stimulus types, and two stranger-associated audiovisual stimulus types. There were 40 trials per condition. Sixteen practice trials preceded the main blocks with a performance threshold set at 80% accuracy – participants could proceed to the main blocks once their performance (responding within 300 ms of stimulus onset) reached or exceeded 80% accuracy. To discourage anticipatory responses, “Too early” feedback was presented if the participant responded during the presentation of the fixation cross and “too slow” was displayed if they responded outside of the time limit, or not at all (such responses were also recorded as incorrect and excluded from the analysis). If participants responded correctly within the time-limit, no feedback was presented. (After the computer tasks, a sub-group of participants completed questionnaires measuring individual differences on self- and other-related dimensions (e.g., ‘personal distance’; Sui & Humphreys, 2015). The data from these instruments will be analysed and presented as part of a separate future study.

### Data analysis

#### Matching tasks

There were two main output measures: RTs (measured from stimulus onset to the depression of the keyboard key), and percentage of correct responses (accuracy). Following previous research (Sui et al., 2012), a signal detection approach was used to calculate an index of sensitivity (D-prime; *d*′; Green & Swets, 1966). Hits were coded as *yes* responses to match trials, and false alarms were coded as *yes* responses to mismatch trials with the same shape. Mismatch conditions were defined as either shape- or sound-based (i.e., a self-mismatch trial consisted of the self-associated shape or sound and the stranger-associated label, a stranger-mismatch trial consisted of the stranger-associated shape or sound and the self-associated label). RTs were based on correct responses. RTs above or below 2.5 SDs from individual means were trimmed (less than ~1% of RTs were excluded). For absolute measures of RT, percentage accuracy, and *d*′ data, see Table 1.

Self-bias index scores were calculated using RTs and *d*′. Normalized self-bias scores in RTs were calculated using the matching-condition RTs (e.g., Constable et al., 2021; Desebrock & Spence, 2021; Sui & Humphreys, 2017a). Match-trial stimuli (e.g., a self-associated object and self-associated label) involve one association, thus in behavioural paradigms where effects in mismatch trials cannot be disentangled, matching-trial responses index self- and stranger-related processing (Sui et al., 2012). Mismatch trials are typically treated as fillers in the literature on the SPE (see Desebrock & Spence, 2021; Schäfer et al., 2016b). Therefore, in accordance with the aims of the present study, and following the rationale of previous research, the focus of the analysis reported here was on match-trial RT data. Self-bias scores were given by the formula: “(stranger − self)/(stranger + self)” for RTs. For *d*′, self-bias was indexed by the differential scores (self – stranger) between self-associated and stranger-associated conditions (following Sui & Humphreys, 2017a). Positive values indicate an advantage for self. (NB: Since auditory stimulus intensity influences RTs and *d*′, main effects of dB level on RT and *d*′ across Groups 1 and 2 were assessed. There was no main effect of auditory stimulus intensity on RTs or *d*′ (see Appendix 2), therefore *d*′ self-bias index scores were not normalised.)

Normalised differential scores – self-bias index scores (e.g., Constable et al., 2021; Desebrock & Spence, 2021; Schäfer et al., 2016b; Sui & Humphreys, 2017a) – were analysed to examine whether the magnitude of the self-advantage in the matching task responses was modulated by stimulus type, block type, and auditory stimulus intensity. These scores provide an index of the relative magnitude of the difference in performance between self- and stranger-related responses. The present study follows previous studies that have examined contextual factors (both stimulus- and task-design-related) that moderate the well-established self-advantage in the matching task using different task parameters (e.g., Golubickis et al., 2017; Golubickis & Macrae, 2021; Hu et al., 2020; Stolte et al., 2021; Verma et al., 2021; Woźniak & Knoblich, 2019). The hypotheses of the present study thus spoke to the modulation rather than the emergence of the SPE in the matching paradigm, so the focus was on analysing differences in the magnitude of the SPE rather than absolute RTs (e.g., see Constable et al., 2021). See Appendix 1 for the analyses of absolute RTs and *d*′ measures. A significant advantage for self-associated responses was found in both the Group 1 (η_p_^2^ = .62) and Group 2 samples (η_p_^2^ = .23) (Appendix 1).

To further assess the self-advantage in responses to unisensory as compared with multisensory stimuli in the matching task, multisensory gains/costs in RTs and sensitivity (*d*′) in the self- and stranger-associated matching conditions were compared. Multisensory facilitation in both the blocked and the intermixed trials in the matching tasks was assessed in RTs and *d*′ by subtracting the RTs/*d*′ scores of responses in the multisensory conditions from the fastest/highest of the counterpart unisensory conditions (e.g., Barutchu et al., 2018, 2020). For example, multisensory facilitation in RT for self-associated responses to the A+VL stimulus type was calculated by deducting A+VL self-associated matching-trial RTs from the fastest of the V+VL and A+AL self-associated matching-trial RTs. Multisensory facilitation in RT for self-associated responses to the V+AL stimulus type was calculated by deducting V+AL self-associated matching-trial RTs from the highest of the V+VL and A+AL self-associated matching-trial RTs. Multisensory facilitation in *d*′ scores for self-associated responses to the A+VL stimulus type was calculated by deducting A+VL self-associated condition *d*′ scores from the highest of the V+VL and A+AL self-associated condition *d*′ scores (and then multiplying by -1 so that positive values indicated gains). Multisensory facilitation in *d*′ scores for self-associated responses to the V+AL stimulus type was calculated by deducting V+AL self-associated condition *d*′ scores from the highest of the V+VL and A+AL self-associated condition *d*′ scores (and then multiplying by −1 so that positive values indicated gains). Thus, the multisensory gain value was the difference between the multisensory and unisensory responses. A positive value indicated faster RTs/higher *d*′ and a negative value indicated slower RTs/lower *d*′ (multisensory costs) for multisensory self- or stranger-associated responses as compared with the fastest/highest unisensory self- or stranger-associated responses. (NB: Mean SPE gains were also calculated but not analysed; they are depicted in Fig. 5c. SPE gains were calculated by deducting the self-associated multisensory condition – Matching- or Mismatching-trial – RTs from the stranger-associated multisensory condition RTs. Positive values indicate a self-advantage.) In order to compare multisensory gains/costs in RTs across Group 1 and Group 2, the multisensory gain/cost measures were converted into percentage gains/costs to control for differences in processing speed, and were given by the formula: [(faster of unisensory – multisensory)/faster of unisensory)] × 100.

#### Detection task

RTs < 100 ms and > 2.5 SDs above individual means were trimmed, excluding < 0.5% of total RTs. Percentage detection rates, RTs, and central tendencies of the RT distributions were examined. There were two within-participant factors: Association on two levels (self, stranger) and Condition with four levels (Auditory, Visual, AV Mismatch, AV Match). Cumulative Distribution Frequency plots (CDFs; Ratcliff, 1979) were also constructed using CDF-XL (Houghton & Grange, 2011) to further assess distributional information and examine differences throughout the whole RT distributions. Percentiles (deciles) for the rank-ordered RTs by condition, for each participant, were calculated. For each condition, cumulative probabilities were calculated from 0.1 up to 1.0 in increments of 0.10. The CDF plots were then drawn using MATLAB. To preserve power, every second probability was included in the analyses. There were three within-participant factors: Association on two levels (self, stranger), Condition on four levels (A, V, AV Mismatch, and AV Match), and Probability on five levels (0.1, 0.3, 0.5, 0.7, 0.9).

Central tendencies were similarly calculated for MS gains (calculated by subtracting the RTs of responses in the multisensory conditions from the fastest of the counterpart unisensory conditions). There were two within-participant factors: Association on two levels (self, stranger) and AV Condition on two levels (Match, Mismatch).

Effect sizes were calculated using Cohen’s *dz* for t-tests and partial eta-squared (η_p_^2^) for ANOVAs (Cohen, 1988; Lakens, 2013). To adjust for multiple comparisons, Holm-Bonferroni corrections at an α value of .05 were applied (Holm, 1979) with unadjusted significance values reported. For violations of sphericity, Greenhouse-Geisser correction were applied where appropriate.

## Results

### Matching tasks

**Fig. 3:**
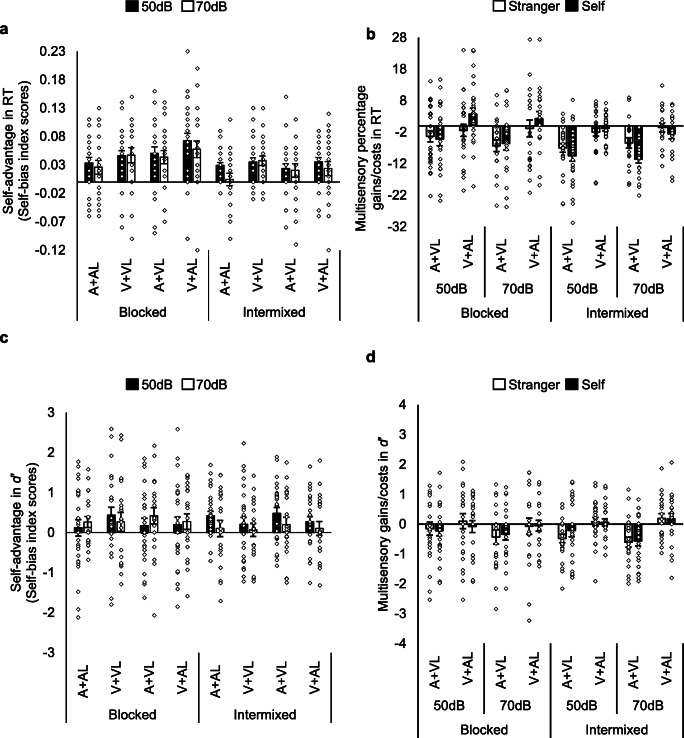
Bar graphs with individual data points (outliers excluded). Error bars represent SE. **a** Selfbias in RT index scores in the shape-label Matching trials as a function of Block type (blocked vs intermixed), Stimulus type, and Auditory stimulus intensity. **b** Multisensory percentage gains/costs in RT as a function of Block type, AV stimulus type, Association, and Auditory stimulus intensity. **c** Selfbias in sensitivity index scores (*d*′; D-prime) as a function of Block type, Stimulus type, and Auditory stimulus intensity. **d.** Multisensory gains/costs in *d*′ as a function of Block type, AV stimulus type, Association, and Auditory stimulus intensity. V = visual shape stimulus, A = auditory stimulus, VL = visual (text) label, AL = auditory (spoken) label

In the 50 dB group (Group 1), the data from two participants were excluded for having chance accuracy (M < 50%) and (< 30% correct in more than one condition). The data were assessed for outliers in the match-trial and mismatch-trial RT data, and the *d*′ data (studentized residuals outside ∓ 3.0 in absolute value in one or more conditions), and one participant’s data was identified as an outlier and thus removed from the analysis (mean RTs and *d*′ data are presented in Table 1). In the 70-dB group (Group 2), five participants were excluded for having chance levels of accuracy, and the data from two participants were excluded as they constituted outliers. The data from a total of 50 participants (28 in Group 1 and 22 in Group 2) were therefore used in the analysis. Self-bias index scores for RT and *d*′, and multisensory RT, *d*′, gains and costs, are presented in Fig. 3

#### Self-bias in reaction times (RTs)

**Fig. 4:**
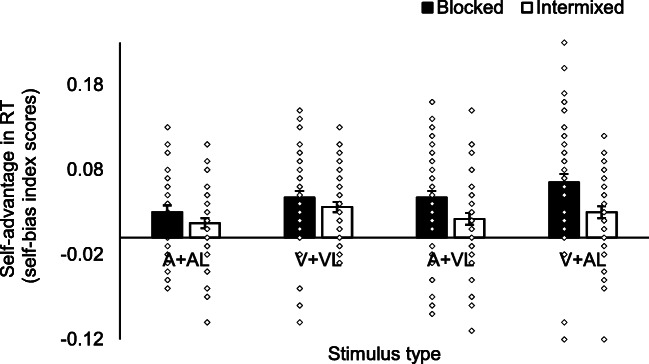
Bar graphs with individual data points (outliers excluded). Estimated marginal means of self-bias in RT index scores in shape-label Matching trials as a function of Block type and Stimulus type (with the Auditory stimulus intensity condition collapsed) showing the main effects of Block type and Stimulus type, and the borderline-significant interaction between Block type and Stimulus type). Error bars represent SE. V = visual shape stimulus, A = sound stimulus, VL = visual (text) label, AL = auditory (spoken) label

The self-bias in RT data is presented in Fig. 3a. There were two outliers (studentized residuals outside ∓ 3.0 in absolute value). Both outliers were removed (N = 48), and since the majority of the data were normally distributed (two conditions not normally distributed), we chose not to transform the data. The assumption of homogeneity of variances was violated for one condition, as assessed by Levene's test for equality of variances. Given that the sample sizes of the two groups were roughly equal (ratio 1.29) and ANOVA is generally robust to violations of this assumption if group sizes are roughly equal, ANOVA was carried out on the data. A 2 (Auditory stimulus intensity: 50 dB, 70 dB) × 2 (Block type: Blocked, Intermixed) × 4 (Stimulus type: V+VL, A+AL, A+VL, V+AL) mixed ANOVA was conducted on the RT self-bias index scores. The analysis revealed main effects of Stimulus type, *F*(2.21, 101.47) = 4.76, *p* = .009, η_p_^2^ = .09, and Block type, *F*(1, 46) = 24.74, *p* < .001, η_p_^2^ = .35. Overall, the magnitude of self-bias in the Blocked trials (*M* = .05, *SE* = .006) was greater than in the Intermixed trials (*M* = .03, *SE* = .005). For Stimulus type, the magnitude of the normalised self-bias in the RT data was significantly greater (*p* = .002) in responses to V+AL (*M* = .05, *SE* = .007) as compared with A+AL (*M* = .02, *SE* = .006) (see Fig. 4). There were no significant differences between any other pair of Stimulus types (*ps* > .01). There were no other significant effects. (The analysis was also conducted with the outliers included – see Table A1, Appendix 3. The findings were replicated.)

#### Self-bias in sensitivity index scores (D-prime; *d*′)

The self-bias in *d*′ data is presented in Fig. 3b. There were two studentized residuals outliers. Both outliers were removed. The assumption of homogeneity of variances was violated for two conditions. As with the analysis of self-bias in the RT data, given that the group sizes were roughly equal, an ANOVA was carried out on the data (N = 48). A 2 (Auditory stimulus intensity: 50 dB, 70 dB) × 2 (Block type: Blocked, Intermixed) × 4 (Stimulus type: V+VL, A+AL, A+VL, V+AL) mixed ANOVA was conducted on self-bias index scores for *d*′. There were no significant effects. The self-advantage in sensitivity did not differ significantly across the conditions. (The analysis was also conducted with the outliers included – see Table A1, Appendix 3. The findings were replicated.)

#### Multisensory percentage gains/costs in RT

The data are presented in Fig. 3c. There were two studentized residuals outliers whose removal produced a third outlier. With the three outliers removed, the data were submitted to a 2 (Auditory stimulus intensity: 50 dB, 70 dB) × 2 (Block type: blocked, intermixed) × 2 (AV Stimulus type: A+VL, V+AL) × 2 (Association: self, stranger) mixed ANOVA. There was a significant main effect of Block type, *F*(1, 45) = 8.85, *p* = .005, η_p_^2^ = .16, and AV Stimulus type, *F*(1, 45) = 50.14, *p* < .001, η_p_^2^ = .53. Negative gain values indicated that there were costs for responses to multisensory relative to unisensory stimuli. Multisensory costs were greater in participants’ responses to intermixed trials (*M* = -4.75, *SE* = 0.66) as compared with blocked trials (*M* = -1.96, *SE* = 1.05), and greater in responses to the A+VL stimulus type (*M* = -6.34, *SE* = 0.88) as compared with responses to the V+AL stimulus type (*M* = -1.37, *SE* = 0.83). There was a significant interaction between Block type and Association, *F*(1, 45) = 8.04, *p* = .007, η_p_^2^ = .15, and between AV Stimulus type and Association, *F*(1, 45) = 5.07, *p* = .03, η_p_^2^ = .10. None of the other effects was significant. The interaction between the AV stimulus type and Association was probed revealing a significant difference between stranger-associated responses to the A+VL (*M* = -5.38, *SE* = 1.02) and V+AL (*M* = -1.44, *SE* = 0.99) stimulus types, *p* = .003, and a significant difference between self-associated responses to the A+VL (*M* = -7.30, *SE* = 1.13) and V+AL stimulus types (*M* = 0.70 ms, *SE* = 0.97), *p* < .001. There was no significant difference between self- and stranger-associated responses to the A+VL stimulus type, *p* = .13, or to the V+AL stimulus type, *p* = .05. However, it is worth noting that for A+VL stimuli, costs were descriptively greater for the self-associated than for the stranger-associated responses, while for the V+AL stimuli, costs were observed for the stranger-associated responses, and gains were uniquely observed for self-associated responses. Probing the Block type and Association interaction revealed greater multisensory costs for self-associated responses (*M* = -5.78, *SE* = 0.80) than for stranger-associated responses (*M* = -3.71, *SE* = 0.78) in intermixed trials, *p* = .02. There was no significant difference between self-associated responses (*M* = -0.82, *SE* = 1.24) as compared with stranger-associated responses (*M* = -3.11, *SE* = 1.18) in blocked trials, *p* = .07. However, costs were descriptively greater in stranger-associated as compared with self-associated responses. In addition, stranger-associated responses in blocked trials (*M* = -3.11, *SE* = 1.18) as compared with intermixed trials (*M* = -3.71, *SE* = 0.78) were not significantly different, *p* = .63. In contrast, multisensory costs in self-associated responses in blocked trials (*M* = -0.82, *SE* = 1.24) as compared with intermixed trials (*M* = -5.78, *SE* = 0.80) were significantly different, *p* < .001. Multisensory costs in self- as compared with stranger-associated responses were differentially modulated by block type. (The analysis was also conducted with the outliers included – see Table A2, Appendix 3. The findings were replicated except that there was no significant difference between self- and stranger-associated responses in intermixed trials, *p* = .06.)

#### Multisensory gains/costs in sensitivity index scores (D-prime; *d*′)

The data for multisensory gains/costs in *d*′ are presented in Fig. 3d. There were four studentized residuals outliers. With the outliers excluded, the data were submitted to a 2 (Auditory stimulus intensity: 50 dB, 70 dB) × 2 (Block type: blocked, intermixed) × 2 (AV stimulus type: A+VL, V+AL) × 2 (Association: self, stranger) mixed ANOVA. The analysis revealed a significant main effect of AV Stimulus type, *F*(1, 44) = 18.30, *p* < .001, η_p_^2^ = .29. Negative gain values indicated that there were costs for responses to multisensory relative to unisensory stimuli. There was some multisensory facilitation in responses to the V+AL stimuli (*M* = 0.06, *SE* = 0.08), and costs in responses to A+VL stimuli (*M* = -0.36, *SE* = 0.08). There was a significant interaction between Block type and AV stimulus type, *F*(1, 44) = 6.21, *p* = .02, η_p_^2^ = .12. There were no other significant effects. The interaction between Block type and AV stimulus type was probed, revealing that costs in responses to the A+VL stimulus type (*M* = -0.28, *SE* = 0.13) as compared with the V+AL stimulus type (*M* = -0.02, *SE* = 0.13) in blocked trials were not significantly different (*p* = .05). There were multisensory gains in responses to the V+AL stimulus type (*M* = 0.14, *SE* = 0.08) as compared with multisensory costs in responses to the A+VL stimulus type (*M* = -0.45, *SE* = 0.09) in the intermixed trials (*p* < .001). (The analysis was also conducted with the outliers included – see Table A2, Appendix 3. The findings were replicated, except that there was a significant difference between multisensory costs in responses to the A+VL and V+AL stimulus types.)

### Detection task

We then examined, for the first time, whether the SPE can transfer to a simple detection task whereby, in contrast to the matching task, the self-associations were irrelevant to the task at hand (Orellana-Corrales et al., 2021; Stein et al., 2016; Woźniak & Knoblich, 2021). After completing the matching task, the participants in Group 2 made motor RT responses (single keypress) irrespective of the stimuli presented (Hecht et al., 2008; Miller, 1982; Wundt, 1910), which consisted of unisensory and multisensory combinations of the self- and stranger-associated auditory objects and visual shapes allocated to the participant in the matching task (without the labels). Completing the blocked and then intermixed trials of the matching task ensured that the participants were equally familiar with all stimulus modality types before completing the detection task.

**Fig. 5:**
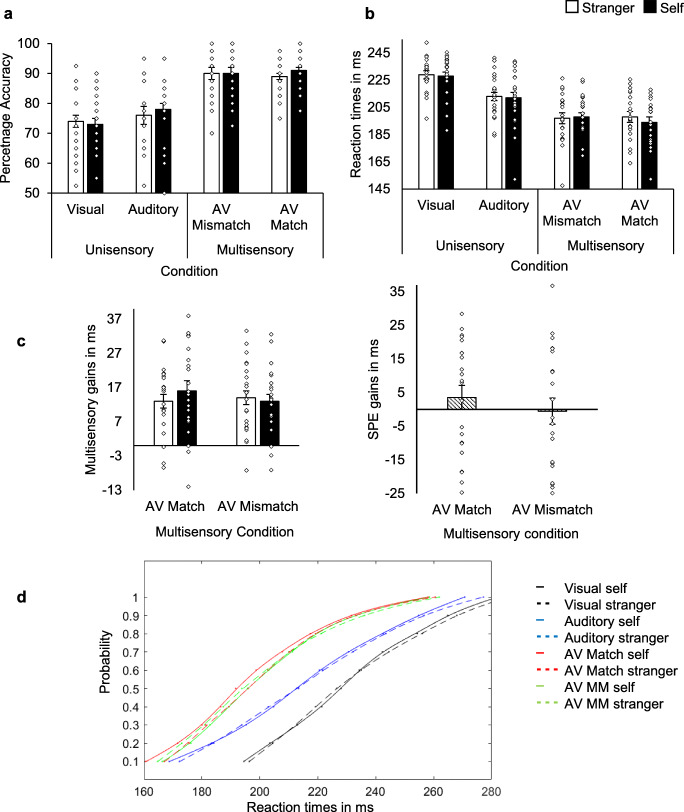
Detection task. N = 22 (Group 2). Bar graphs (**a**-**c**) with individual data points (outliers excluded). Error bars represent SE. **a** Mean Percentage Accuracy (detection rate) as a function of Association and Stimulus type. **b** Mean RTs as a function of Association and Condition (Stimulus type). **c** Mean multisensory RT gains in ms as a function of Association and Multisensory Condition (left). Mean SPE RT gains in ms as a function of multisensory condition (right). SPE gains = the difference between self- and stranger-associated responses. Positive values indicate a self-advantage. **d** Cumulative Distribution Functions (CDFs) of percentiles of the rank-ordered RTs (PC grand RT means) as a function of Condition (uni- and multisensory). AV Match self = the sound and shape associated with the self in the preceding matching task. AV Match stranger = the sound and shape associated with the stranger in the preceding matching task. MM = mismatch. AV self MM = self-associated shape and stranger-associated sound used in the preceding matching task. AV MM stranger = stranger-associated shape and self-associated sound used in the preceding matching task

The data from four participants were excluded for achieving > 2.5 SDs below the group percentage detection rate mean for any one condition, or, for achieving < 50% accuracy in any one condition. The data from 22 participants were used in the analysis. Percentage detection rate data, RT data, CDFs of RTs, and multisensory RT gains are all presented in Fig. 5

### Accuracy

For the detection rate data, see Fig. 5a. The studentized residuals for one condition were not normally distributed. Since the majority of the data were normally distributed, we chose not to transform the data. Percentage detection rate scores were submitted to a 2 (Association: Self, Stranger) × 4 (Stimulus type: Visual, Auditory, AV Mismatch, AV Match) repeated-measures ANOVA. There was a significant main effect of Stimulus type, *F*(3, 63) = 50.15, *p* < .001, η_p_^2^ = .71. There were no other significant effects. Pairwise comparisons between stimulus types revealed that detection rates in matching (*M* = 89.77, *SE* = 1.27) and mismatching (*M* = 89.77, *SE* = 1.63) multisensory trials were significantly greater than in unisensory visual (*M* = 73.35, *SE* = 2.23) and unisensory auditory (*M* = 77.39, *SE* = 2.53) trials (*p*s < .001). There was no significant difference between AV Mismatch and AV Match Stimulus types (*p* > .99), or between Auditory and Visual conditions (*p* = .05; unadjusted significance value reported, Holm-Bonferroni correction, see *Data analysis*). Detection rates on AV trials were significantly higher than detection rates in unisensory trials.

### RT

One condition was not normally distributed. Since the majority of the data were normally distributed, we chose not to transform the data. RTs were submitted to a 2 (Association: Self, Stranger) × 4 (Stimulus type: Visual, Auditory, AV Mismatch, AV Match) repeated-measures ANOVA. There was a significant main effect of Stimulus type, *F*(2.20, 46.23) = 115.95, *p* < .001, η_p_^2^ = .85 (Greenhouse-Geisser correction). There were no other significant effects. Pairwise comparisons between stimulus types revealed a significant difference between all stimulus types (*ps* < .001) except for between AV Mismatch and AV Match conditions (*p* = .14). Responses in AV trials were faster than in unisensory trials, and responses in the Auditory trials were faster than in the Visual trials (see Fig. 5b).

### Multisensory facilitation in RT

Multisensory gain/cost values (faster of unisensory – multisensory) were submitted to a 2 (Association: self, stranger) × 2 (AV stimulus type: match, mismatch) repeated-measures ANOVA. There were no significant effects (Fig. 5c, left graph). SPE gains (see *Data analysis*) can also be seen in Fig. 5c (right graph). Multisensory percentage gains/costs were also calculated, the analysis re-run, and the findings were replicated.

### CDFs: RTs

Three of the forty conditions (2 Association × 4 Stimulus type × 5 Probabilities) were not normally distributed, and there was one studentized residuals outlier. With the outlier excluded, studentized residuals for two of the 40 conditions were not normally distributed. Since the majority of the data were normally distributed, we chose not to transform the data. With the outlier excluded (N = 21), RTs were submitted to a 2 (Association: self, stranger) × 4 (Stimulus type: A, V, AV Mismatch, AV Match) × 5 (Probability: 1, 3, 5, 7, 9) repeated-measures ANOVA. There were main effects of Stimulus type, *F*(2.17, 43.40) = 117.55, *p* < .001, η_p_^2^ = .86, and Probability, *F*(1.34, 26.70) = 773.53, *p* < .001, η_p_^2^ = .98. There was a significant interaction between Stimulus type and Probability, *F*(5.48, 109.63) = 9.13, *p* < .001, η_p_^2^ = .31. (Greenhouse-Geisser correction used for all effects with more than two factors.) There were no other significant effects. Pairwise comparisons for Stimulus type revealed significant differences between the unisensory and multisensory conditions (*ps* < .001), and the auditory (*M* = 215.17 ms, *SE* = 2.28) and visual conditions (*M* = 230.79 ms, *SE* = 2.25), (*ps* < .001), but no significant difference between AV Mismatch (*M* = 199.37 ms, *SE* = 2.42) and AV Match (*M* = 197.84 ms, *SE* = 2.69) conditions, *p* = .11 (see Fig. 5d). (The analysis was also conducted with the outlier included. The findings were replicated.)

The participant data exclusion criteria for the detection task analysis (N = 22 sample) were based on participant performance in the detection task (irrespective of their performance in the matching task). This could potentially confound the transfer of the social associations because any participants with lower performance in the matching task may not have properly integrated the associations for access during the detection task. To check whether performance in the matching task could have impacted the influence of self-associations in the detection task, a supplementary analysis of RTs and multisensory percentage gains was conducted. This time the analysis excluded participant data that had been excluded from both the matching and the detection task analyses combined (N = 16; see Appendix 4). The findings replicated the findings with the N = 22 sample.

## Discussion

Using an audiovisual adaptation of Sui et al.’s (2012) matching task, the present study investigated the SPE – the magnitude of the difference in performance between motor responses to self-associated and stranger-associated stimuli. The SPE was modulated by whether the simultaneously presented label and object pairs were visual, auditory, or a combination of the two modalities. Specifically, the SPE in RT was significantly greater in responses to the visual shape and auditory label stimuli (V+AL) than to the auditory object and auditory label (A+AL) stimuli, and there was a significant interaction between association (self- vs. stranger-associated responses) and audiovisual stimulus type (A+VL vs. V+AL) for multisensory gains/costs in RT. Multisensory costs were descriptively greater for self- than stranger-associated A+VL stimuli, while for the V+AL stimuli, there were costs for stranger, and gains were uniquely observed for self. As such, the SPE interacted with the combination of the object and label stimulus modalities. The SPE in RT was also diminished when stimuli were intermixed as compared with blocked by stimulus modality type. Furthermore, no significant self-advantage was found in simple detection task motor responses to the unisensory and multisensory stimuli in which the learnt self-associations were not relevant to the task at hand. Taken together, the present findings therefore indicate that the SPE can be modulated by both stimulus- and task-related parameters within the matching task, but the self-associations formed in the matching task do not automatically result in similar motor speed gains to unisensory and multisensory stimuli in fast (< 300 ms) simple RT motor responses.

### SPE in the matching task

The present study findings are both consistent with, and also depart from, the findings reported by previous research. It was hypothesized that the SPE would be moderated by block type. Consistent with this prediction, and previous research (Golubickis & Macrae, 2021) block type moderated the SPE in RT. The SPE was reduced in intermixed trials (cf. the increased SPE in intermixed trials in the previous study in which visual stimuli were blocked or intermixed by the target shape identity). One possibility is that increasing fatigue may have reduced the SPE in intermixed trials since the intermixed condition was presented after the blocked condition. Conversely, the increased familiarity of the stimuli and with the S-R mappings gained in the blocked trials might be expected to increase the SPE in intermixed trials. An additional analysis was conducted, however, to examine the magnitude of the SPE across individual blocks within the blocked and intermixed conditions. The analysis confirmed that the SPE did not significantly decrease within either the blocked or the intermixed condition (see Appendix 6). Multisensory costs in self- as compared with stranger-associated responses were also differentially modulated by block type in the present study. Costs in RT were significantly reduced in blocked as compared with intermixed trials for self-associated responses, whereas costs in stranger-associated responses across block types did not differ. Taken together, these finding suggest that self- and stranger-associated responses were differentially modulated by block type.

It was also hypothesized, should findings be consistent with the pattern of results across previous studies examining the SPE using visual and auditory stimuli (Schäfer et al., 2016b; Stolte et al., 2021), that the SPE would be reduced with audiovisual as compared with visual stimuli, but that the latter may depend on the block type and the auditory stimulus intensity. Specifically, using intermixed trials and auditory stimuli presented at 75 dB, Stolte et al. (2021) documented that the SPE was diminished in those responses made to audiovisual stimuli (i.e., auditory tones with visual labels, and particularly diminished in the absence of labels with audiovisual objects) as compared with visual-only stimuli with labels. Meanwhile, using blocked trials and auditory stimuli presented at 50 dB, Schäfer et al. (2016b) found that the SPE was of equivalent magnitude in responses to visual, auditory, and tactile stimuli paired with visual labels. In the present study, the SPE in RT was significantly attenuated with auditory as compared with visual objects (consistent with Stolte et al., 2021), but only when paired with auditory labels. (NB. Stolte et al. only paired their stimuli with visual labels.) Furthermore, there was an interaction between association (self- vs. stranger-associated responses) and audiovisual stimulus type (A+VL vs. V+AL) for multisensory gains/costs in RT. The multisensory (A+VL) stimuli were associated with ‘costs’ rather than gains over unisensory stimulation (in line with our expectations, and consistent with Stolte et al., 2021), with descriptively greater costs for self- than stranger-associated responses. However, while costs were also observed in participants’ responses to stranger-associated multisensory (V+AL) stimuli, multisensory gains were observed in responses to the self-associated multisensory (V+AL) stimuli. Thus, the present study provides the first evidence that the combination of the label and object modality (auditory vs. visual) can modulate the SPE.

Taken together, these patterns of results suggest that the SPE is influenced by both the task design (blocked vs. intermixed trials) and the stimulus parameters (the modality of the object and label stimuli). Contrary to predictions, however, auditory stimulus intensity did not moderate the SPE in the present study. Further analyses confirmed that there were no significant main nor interaction effects of auditory stimulus intensity on the SPE in the absolute RTs either – analysis reported in Appendix 5. It is important to note that a very narrow super-threshold dB range (50 dB and 70 dB) was used in order to replicate past studies (Schäfer et al., 2016b; Stolte et al., 2021). In future studies, comparing the SPE across a greater range of auditory stimulus intensities from near-threshold levels to upper limits may yield different results.

It was also hypothesized that a reduced SPE with audiovisual stimuli would lend support to Stolte et al.’s (2021) findings, whereas an equivalent SPE across stimulus modality types would lend support to the contention that the SPE is underpinned by a modulation of modality-general processes (in line with Schäfer et al., 2016b). In their study, Stolte et al. also demonstrated that the SPE and, in turn, visual dominance, was modulated by the relative physical hierarchy of the tones used (i.e., the multisensory SPE was akin to the visual SPE if the high tone was paired with the self). The authors suggested that it may, under certain conditions, be easier to form self-associations with visual representations of objects than audiovisual representations. Meanwhile, Schäfer et al. (2016b) have suggested that the SPE is likely to be a modality-general mechanism, since the SPE was of equivalent magnitude in responses to their visual, auditory, and tactile stimuli paired with visual labels. As noted, the present study found that the magnitude of the SPE in the matching task was moderated across stimulus modality types, but was not consistently reduced with auditory stimuli. Thus, we did not find evidence to support the notion that the SPE is underpinned by modality-general processes (although this could not be ruled out), nor that it may be easier to form visual self-associations per se.

Schäfer et al.’s (2016b) findings and the results of the present study suggest that when the visual label is paired with an auditory or visual object, the self-advantage that arises is of equivalent magnitude. However, the use of auditory labels paints a rather different picture. In the present study, a significant difference between the SPE in responses to the A+AL and V+AL stimuli was documented. One interpretation for this asymmetry is that self-reference has the most marked advantage over stranger-reference in forming or accessing multisensory associations (specifically involving the visual shape and auditory label stimuli), and the weakest advantage in forming or accessing unisensory auditory associations. Whether self-associations involve sensory or modality-general representations or both is a question that is outside the scope of the present study (cf. Schäfer et al., 2020b). At the very least, when visual or auditory stimulus objects are paired with auditory labels, the present study suggests that the SPE is reduced with auditory stimuli.

Task-related factors, for example, that elicit active attendance to visual over auditory stimuli, could also moderate the SPE across stimulus modality types. As noted, one possibility is that the alerting properties of auditory stimuli may elicit an override response in participants such that they actively focus their attention toward the weaker visual stimuli as a consequence. Fewer cognitive resources thus remain to attend to, and process, auditory stimuli (Posner et al., 1976). We also tend to rely more on visual than auditory information (Sinnett et al., 2007) and visual dominance is socially and culturally reinforced (Hutmacher, 2019). Parsing and extracting the semantic content of simultaneously-presented auditory object and label stimuli versus visual object and auditory label stimuli is more unusual. Cues to solve the former task may automatically be sought from the visual system, for example, modulating auditory stimulus processing, and thus the SPE. Determining the strategies underlying the processing of the object-label stimuli in the A+AL and V+AL conditions (e.g., using imaging methods such as functional magnetic resonance imaging (MRI) and electroencephalography (EEG)), and examining how self-relevance interacts with these processes, would likely provide further insight into the mechanisms underlying the SPE.

### SPE in the detection task

Task instructions and whether the meaning of the stimuli are relevant to the task at hand can also modulate both self-bias and multisensory processes (Barutchu & Spence, 2021; Caughey et al., 2021; Dalmaso et al., 2019; Falbén et al., 2019; Macrae et al., 2017; Woźniak & Knoblich, 2021). Indeed, in contrast to the matching task, the predicted ‘classic’ multisensory gain was consistently observed in the detection paradigm, but no statistically significant self-advantage (i.e., SPE) was detected in either RTs or gain measures. Neural processes can rapidly change in anticipation of task-relevant stimuli in visual tasks (Corbetta et al., 2000; Nobre & van Ede, 2018; Stokes et al., 2009), and task instructions have been shown to alter multisensory gains and costs across consecutive tasks (Barutchu & Spence, 2021; Sinnett et al., 2008). Such top-down processes determined by task instruction may interact with the SPE and multisensory processes to modulate gains and costs to task-relevant and -irrelevant stimuli.

The absence of a significant SPE in the detection task, however, may also be related to other factors. The RT gains were much smaller than expected (on average in the order of 20 ms) as compared with past studies (e.g., Barutchu et al., 2009; Miller, 1982). Unlike in prior studies in which responses are typically limited to an 800- to 2,000-ms window, RTs were limited to 300 ms. This short response time limit was used to ensure that participants did not delay their responses (to avoid the intentional evaluation of the stimuli), and to keep participants alert to the task and responding as fast as they could, rather than simply instructing them to respond as fast as possible. This limited response window, and pressure to respond as rapidly as possible, may have reduced the RT gain measures.

Participants in Group 2 may also have been fatigued, with attention and motivation levels low during the detection task as it was always performed last to allow for optimal learning of stimulus associations. Multisensory processes and facilitation effects are partly dependent on attention (e.g., Barutchu et al., 2021; Talsma, 2015; Talsma et al., 2010; Zuanazzi & Noppeney, 2019). Our findings, however, are consistent with previous studies using other task paradigms that did not observe self-prioritization when the stimuli were not semantically evaluated (Caughey et al., 2021; Dalmaso et al., 2019; Falbén et al., 2019; Stein et al., 2016), or at least when the associations were not represented by the participant as task relevant (Woźniak & Knoblich, 2021). Furthermore, as noted earlier, additional analyses (see Appendix 6) suggested that the SPE did not significantly decrease across individual blocks within either the blocked condition or the intermixed condition in the preceding Matching task.

One further consideration is that in the present study geometric shapes were used as visual stimuli, while the sound stimuli were musical instrument bursts, in order to keep the stimuli consistent with previous research (Schäfer et al., 2016b; Stolte et al., 2021). Alternatively, drawings of musical instruments could be used as visual stimuli (i.e., a different exemplar of the same category; Schäfer et al., 2015). Using different categories across visual and auditory stimuli may have amplified the working memory load in intermixed trials and further reduced the SPE in the present study. Notably, however, if increased working memory load reduces the SPE, then a reduced SPE would still be expected in intermixed trials even with conceptually more similar stimuli. Furthermore, the short 150-ms bursts were not typical of the characteristic sounds made by those instruments, and although recognisable as belonging to their respective categories (when narrowed down to two possibilities), the sound bursts were not typical of the instruments or familiar. This would be more likely to facilitate the forging of self/other associations with sensory rather than conceptual features of the sounds. Conversely, using drawings of the instruments and sounds of the instruments would encourage the use of ‘conceptual’ associations as this would be the optimal strategy to maximise responding across modality types in intermixed trials. Future studies could examine whether matching the semantic content of the stimuli (other than the person associations) across stimulus types modulates the SPE in intermixed trials.

## Conclusion

The present study found that the SPE in an audiovisual adaptation of Sui et al.’s (2012) matching task was diminished in intermixed as compared with trials blocked by stimulus modality type, and interacted with label and object modality (auditory or visual). The standard-sized self-advantage did not arise in simple detection motor responses to the unisensory and multisensory stimuli. Taken together, these findings indicate that the SPE is modulated by both stimulus- and task-related parameters within the matching task, and that the self-associations formed in the matching task do not automatically result in similar motor speed gains to unisensory and multisensory stimuli in a simple detection task.

### Supplementary Information

ESM 1 (PDF 294 kb)

ESM 2 (PDF 316 kb)

## Appendix 1

**Absolute RTs and sensitivity index scores (D-prime;** ***d*****′) (Group 1).** There was one studentized residuals outlier (outside ∓ 3.0 in absolute value in one or more of the conditions) in RT. With the outlier removed (N = 27), the matching-trial RT data were submitted to a 2 (Block type: blocked, intermixed) × 4 (Stimulus type: V-shape+VLabel, A+AL, A+VL, V+AL) × 2 (Association: Self, Stranger) repeated-measures ANOVA. The analysis revealed significant main effects of Block type, *F*(1, 26) = 191.85, *p* <.001, η_p_^2^ = .88, Stimulus type, *F*(2.186, 56.849) = 22.61, *p* < .001, η_p_^2^ = .47, and Association, *F*(1, 26) = 43.07, *p* < .001, η_p_^2^ = .62. RTs were shorter in the Blocked (*M* = 705 ms, *SE* = 9.24) as compared with the Intermixed trials (*M* = 781 ms, *SE* = 9.49). There were significant differences in RTs between all Stimulus types (*ps* < .004), except between V+VL and V+AL (*p* = .10). RTs in self (*M* = 715 ms, *SE* = 9.58) as compared with stranger-associated trials were shorter (*M* = 771 ms, *SE* = 10.26), indicating the presence of a SPE.

There was a significant interaction between Block type and Stimulus type, *F*(3, 78) = 8.55, *p* < .001, η_p_^2^ = .25. There were no other significant interaction effects. Probing the interaction between Block type and Stimulus type revealed a significant difference between blocked and intermixed trial responses across all stimulus types (*ps* < .001). RTs were shorter in the blocked as compared with the intermixed stimulus type trials. In the blocked trials, only the difference in RTs between A+AL and V+AL was significant (*p* < .001). In contrast, in intermixed trials, there was a significant difference between each of the stimulus types (*ps* < .001) except for between V+VL and V+AL (*p* = .34). (The analysis was conducted again with the outlier included and the findings were replicated.)

The *d*′ data were normally distributed except for one condition (Blocked V+AL Stranger), therefore we chose not to transform the data. Sensitivity index scores were submitted to a 2 (Block type: blocked, intermixed) × 4 (Stimulus type: V-shape+VLabel, A+AL, A+VL, V+AL) × 2 (Association: Self, Stranger) repeated-measures ANOVA. There were significant main effects of Block type, *F*(1, 27) = 29.26, *p* < .001, η_p_^2^ = .52, Stimulus type, *F*(2.183, 58.943) = 15.67, *p* < .001, *ηp*^2^ = .37 (Greenhouse-Geisser correction), and Association, *F*(1, 27) = 12.89, *p* = .001, η_p_^2^ = .32. *d*′ scores were higher for responses to Blocked (*M* = 2.56, *SE* = .09) as compared with Intermixed (*M* = 1.94, *SE* = .14) trials. There were significant differences between all Stimulus types (*ps* < .01 for all) except for between V+VL and A+VL (*p* = .76). *d*′ scores were higher for self- (*M* = 2.38, *SE* = .12) as compared with stranger-associated (*M* = 2.11, *SE* = .11) responses, indicating the presence of a SPE.

**Absolute RTs and sensitivity index scores (D-prime;** ***d*****′) (Group 2).** There was one studentized residuals outlier (outside ∓ 3.0 in absolute value) in RT. With the outlier removed (N = 21), studentized residuals were normally distributed except for in two of the conditions. Since the majority of the data were normally distributed, we chose not to transform the data. The matching trial RT data (N = 21) were submitted to a 2 (Block type: blocked, intermixed) × 4 (Stimulus type: V+VL, A+AL, A+VL, V+AL) × 2 (Association: Self, Stranger) repeated measures ANOVA. There were significant main effects of Block type, *F*(1, 20) = 53.03, *p* <.001, η_p_^2^ = .73, Stimulus type, *F*(3, 60) = 19.83, *p* < .001, η_p_^2^ = .50, and Association, *F*(1, 20) = 6.05, *p* = .02, η_p_^2^ = .23. In contrast to the results for Group 1, there were also significant simple two-way interactions between Block type and Association, *F*(1, 20) = 9.72, *p* = .01, η_p_^2^ = .33, and between Stimulus type and Association, *F*(3, 60) = 4.30, *p* = .008, η_p_^2^ = .18, and, as for Group 1, between Block type and Stimulus type, *F*(3, 60) = 3.64, *p* = .02, η_p_^2^ = .15. Probing the interaction between Stimulus type and Association revealed a significant difference between self- and stranger-associated responses for the V+VL (*p* = .005, *ηp*^2^=.34) and V+AL (*p* = .008, η_p_^2^ = .30) stimulus types, but not for the A+VL (*p* = .07, η_p_^2^ = .16) and A+AL (*p* = .39, η_p_^2^ = .04) stimulus types. Probing the interaction between Block type and Association revealed a significant difference between self- and stranger-associated responses in the Blocked trials, *F*(1, 21) = 9.20, *p* = .007, η_p_^2^ = .32, but not in the intermixed trials, *F*(1, 21) = 2.64, *p* = .12, η_p_^2^ = .12. Once again, these findings were replicated with the outlier included.

Sensitivity index scores were submitted to a 2 (Block type: blocked, intermixed) × 4 (Stimulus type: V+VL, A+AL, A+VL, V+AL) × 2 (Association: Self, Stranger) repeated-measures ANOVA. The analysis revealed significant main effects of Block type, *F*(1, 21) = 40.61, *p* <.001, ηp^2^ = .66, and Stimulus type, *F*(3, 63) = 14.28, *p* < .001, η_p_^2^ = .41, but, in contrast to Group 1, not of Association, *F*(1, 21) = 1.90, *p* = .18, η_p_^2^ = .08. Self- (*M* = 2.31, *SE* = .16). Self- as compared with stranger- (*M* = 2.15, *SE* = .20) associated *d*′ scores were not significantly different. In contrast to Group 1, there was a significant interaction between Block type and Stimulus type, *F*(3, 63) = 4.95, *p* = .004, η_p_^2^ = .19.

## Appendix 2

**Matching tasks (Groups 1 and 2).** An independent *t*-test was conducted on overall RT across Groups 1 and 2 revealing no significant difference between the 50-dB auditory group (*M* = 742 ms, *SD* = 46 ms) and the 70-dB stimulus intensity group (*M* = 730 ms, *SD* = 54 ms), *t*(48) = 0.82, *p* = .42. Similarly, there was no significant difference between overall *d*′ scores between the 50-dB group (*M* = 2.25, *SD* = 0.56) and the 70-dB group (*M* = 2.23, *SD* = 0.79), *t*(36.31) = 0.07, *p* = .94.

A 2 (Auditory stimulus intensity: 50 dB, 70 dB) × 2 (Block type: Blocked, Intermixed) × 4 (Stimulus type: V+VL, A+AL, A+VL, V+AL) ANOVA on RTs across Groups 1 and 2 with the Association (self, stranger) condition collapsed revealed no effect of auditory stimulus intensity. In particular, there was no interaction between Block type and Auditory stimulus intensity (*p* = .31), nor between Stimulus type and Auditory stimulus intensity (*p* = .57), nor was there a three-way interaction (*p* = 1.00).

A 2 (Auditory stimulus intensity: 50 dB, 70 dB) × 2 (Block type: Blocked, Intermixed) × 4 (Stimulus type: V+VL, A+AL, A+VL, V+AL) ANOVA on *d*′ scores across Groups 1 and 2 with the Association (self, stranger) condition collapsed revealed no effect of auditory stimulus intensity: There was no interaction between Block type and Auditory stimulus intensity, *p* = .90, nor between Stimulus type and Auditory stimulus intensity (*p* = .72), nor was there a three-way interaction (*p* = .44).

## Appendix 3

The following analyses replicate those presented in the main text, but with the outliers included (rather than excluded) in each case. The findings reported in the main text were all replicated.

**Self-bias in RTs with outliers included**

F-statistics are presented in Table A1. There were two outliers (studentized residuals outside ∓ 3.0 in absolute value) identified in the self-bias index scores (there was one outlier which when removed revealed a second outlier). The majority of the data were normally distributed (three conditions not normally distributed), so we chose not to transform the data. The assumption of homogeneity of variances was violated for one condition, as assessed by Levene's test for equality of variances. Given that the sample sizes of the two groups were roughly equal (ratio 1.29) and ANOVA is generally robust to violations of this assumption if group sizes are roughly equal, ANOVA was carried out on the data. With the outliers included, a 2 (Auditory stimulus intensity: 50 dB, 70 dB) × 2 (Block type: Blocked, Intermixed) × 4 (Stimulus type: V+VL, A+AL, A+VL, V+AL) mixed ANOVA was conducted on the RT self-bias index scores. The analysis revealed main effects of Stimulus type, and Block type. Overall, the magnitude of self-bias in the Blocked trials (*M* = .04, *SE* = .007) was greater than in the Intermixed trials (*M* = .02, *SE* = .005). For Stimulus type, the magnitude of the normalised self-bias in the RT data was significantly greater (*p* = .002) in responses to V+AL (*M* = .04, *SE* = .007) as compared with A+AL (*M* = .02, *SE* = .006). There were no significant differences between any other pair of Stimulus types (*ps* > .02). There were no other significant effects. These findings replicated those with the outliers excluded – see main text for details.

**Self-bias in sensitivity index scores (D-prime;** *d*′**) with outliers included**

F-statistics are presented in Table A1. There were two studentized residuals outliers (studentized residuals outside ∓ 3.0 in absolute value) identified in the self-bias index scores (there was one outlier which when removed revealed a second outlier). The assumption of homogeneity of variances was violated for two conditions. As with the analysis of self-bias in the RT data, given that the group sizes were roughly equal, an ANOVA was carried out on the data (N = 50). With the outliers included, a 2 (Auditory stimulus intensity: 50 dB, 70 dB) × 2 (Block type: Blocked, Intermixed) × 4 (Stimulus type: V+VL, A+AL, A+VL, V+AL) mixed ANOVA was conducted on self-bias index scores for *d*′ (see Table 1 – main text). There were no significant effects. The SPE in sensitivity between self- and stranger-associated responses did not differ significantly across the conditions. These findings replicated those with the outliers excluded – see main text.

**Multisensory percentage gains/costs in RT with outliers included**

F-statistics and pairwise comparisons are presented in Table A2. There were two studentized residuals outliers whose removal produced a third outlier. With the outliers included, the data were submitted to a 2 (Auditory stimulus intensity: 50 dB, 70 dB) × 2 (Block type: blocked, intermixed) × 2 (AV Stimulus type: A+VL, V+AL) × 2 (Association: self, stranger) mixed ANOVA. There was a significant main effect of Block type, and AV Stimulus type. Negative gain values indicated that there were costs for responses to multisensory relative to unisensory stimuli. Multisensory costs were greater in participants’ responses to intermixed trials (*M* = -4.99 ms, *SE* = 0.75) as compared with blocked trials (*M* = -1.92 ms, *SE* = 0.98), and greater in responses to the A+VL stimulus type (*M* = -6.72, *SE* = 0.94) as compared with responses to the V+AL stimulus type (*M* = -0.20 ms, *SE* = 0.78). There was a significant interaction between Block type and Association, and between AV Stimulus type and Association. None of the other effects was significant.

Probing the interaction between AV stimulus type and Association revealed a significant difference between stranger-associated responses to the A+VL (*M* = -5.88 ms, *SE* = 1.14) and V+AL (*M* = -1.19 ms, *SE* = 0.94) stimulus types, and a significant difference between self-associated responses to the A+VL (*M* = -7.55 ms, *SE* = 1.10) and V+AL stimulus types (*M* = 0.79 ms, *SE* = 0.92). There was no significant difference between self- and stranger-associated responses to the V+AL stimulus type, or the A+VL stimulus type.

Probing the Block type and Association interaction revealed no significant difference in multisensory costs for self-associated responses (*M* = -5.86 ms, *SE* = 0.78) than for with stranger-associated responses (*M* = -4.13 ms, *SE* = 0.96) in intermixed trials. There was no significant difference between self-associated responses (*M* = -0.90 ms, *SE* = 1.17) as compared with stranger-associated responses (*M* = -2.94 ms, *SE* = 1.11) in the blocked trials, but it is worth noting that costs were descriptively greater in stranger-associated as compared with self-associated responses. In addition, stranger-associated responses in blocked trials as compared with intermixed trials were not significantly different. In contrast, multisensory costs in self-associated responses in blocked trials as compared with intermixed trials were significantly different. Multisensory costs in self- as compared with stranger-associated responses were differentially modulated by block type. These findings replicated those with the outliers excluded – see main text, except that there was no significant difference between multisensory costs for self- versus stranger-associated responses in intermixed trials.

**Multisensory gains/costs in sensitivity index scores (D-prime;** *d*′**) with outliers included**

F-statistics and pairwise comparisons are presented in Table A2. There were four studentized residuals outliers. With the outliers included, the data were submitted to a 2 (Auditory stimulus intensity: 50 dB, 70 dB) × 2 (Block type: blocked, intermixed) × 2 (AV stimulus type: A+VL, V+AL) × 2 (Association: self, stranger) mixed ANOVA. The analysis revealed a significant main effect of AV Stimulus type. Negative gain values indicated that there were costs for responses to multisensory relative to unisensory stimuli. There was some multisensory facilitation in responses to the V+AL stimuli (*M* = 0.06, *SE* = 0.08), and costs in responses to A+VL stimuli (*M* = -0.38, *SE* = 0.08). There was a significant interaction between Block type and AV stimulus type. There were no other significant effects. The interaction between Block type and AV stimulus type was probed revealing that multisensory costs in responses to the A+VL stimulus type (*M* = -0.25, *SE* = 0.13) as compared with the V+AL stimulus type (*M* = 0.01, *SE* = 0.13) in blocked trials were significantly different. There were multisensory gains in responses to the V+AL stimulus type (*M* = 0.10, *SE* = 0.09) as compared with multisensory costs in responses to the A+VL stimulus type (*M* = -0.51, *SE* = 0.11) in the intermixed trials. (These findings replicated those with the outliers excluded – see main text, except that there was a significant difference between multisensory costs in responses to the A+VL and V+AL stimulus types with the outliers included.)

## Appendix 4

**Multisensory simple detection task (Group 2).** The detection task sample included data from those participants whose data were excluded from the preceding matching task because they achieved below-threshold accuracy. This could potentially confound the transfer of the social associations because these participants may not have properly assimilated them. To check whether the low accuracy performance in the matching task could have impacted the influence of self-associations in the detection task, a further analysis was also conducted.

***Participants***

Twenty-six participants completed the detection task. The data from four participants were excluded for having >2.5 SDs below the group percentage accuracy mean for any one condition, or for achieving <50% accuracy in any one condition in the detection task. In addition, the data from the seven participants that were excluded from analyses of the matching task data for low performance accuracy or constituting outliers (see main text) were also excluded. The data from 16 participants were used in the analysis.

***RTs***

The RT data were submitted to a 2 (Association: Self, Stranger) × 4 (Stimulus type: Visual, Auditory, AV Mismatch, AV Match) repeated-measures ANOVA. There was a significant main effect of Stimulus type, *F*(2.05, 30.81) = 105.01, *p* < .001, η_p_^2^ = .88 (Greenhouse-Geisser correction). There was no main effect of Association, *F*(1, 15) = .15, *p* = .70, η_p_^2^ = .01. Pairwise comparisons between stimulus types revealed a significant difference between the unisensory and multisensory stimuli, and between the visual and auditory stimuli (*ps* < .001). Note that the findings using the N = 16 sample thus replicated the findings using the N = 22 sample.

***Multisensory percentage gains/costs***

Multisensory percentage gain/cost values ([(faster of unisensory – multisensory) / faster of unisensory)*100]; see *Data analysis*)) were submitted to a 2 (Association: self, stranger) × 2 (AV stimulus type: match, mismatch) repeated-measures ANOVA. There were no significant effects. The findings using the N = 16 sample thus replicated the findings using the N = 22 sample.

## Appendix 5

Auditory stimulus intensity did not moderate the SPE index scores. To confirm that there were no main nor interaction effects of auditory stimulus intensity on the SPE in the raw RTs, an additional ANOVA was carried out. The majority of the RT data were normally-distributed (two conditions not normally distributed), so we chose not to transform the data. There was one studentized residuals outlier. The assumption of homogeneity of variances was violated for one condition, as assessed by Levene's test for equality of variances. As with the analysis of self-bias in the RT data, given that the group sizes were roughly equal, an ANOVA was carried out on the data. With the outlier included (N = 48), RTs were submitted to a 2 (between-groups Intensity: 50 dB, 70 dB) × 2 (within-groups Block type: blocked, intermixed) × 4 (within groups Stimulus type: V+VL, A+AL, A+VL, V+AL) × 2 (Within-groups Association: self, stranger) mixed factorial ANOVA. The analysis revealed main effects of Stimulus type, *F*(2.46, 113.10) = 40.81, *p* < .001, η_p_^2^ = .47, Block type, *F*(1, 46) = 187.56, *p* < .001, η_p_^2^ = .80, and Association, *F*(1, 46) = 55.09, *p* < .001, η_p_^2^ = .55. There was a significant interaction between Block type and Stimulus type, *F*(3, 138) = 10.10, *p* < .001, η_p_^2^ = .18, Block type and Association, *F*(1, 46) = 15.60, *p* < .001, η_p_^2^ = .25, and Stimulus type and Association, *F*(2.20, 100.97) = 4.34, *p* = .01, η_p_^2^ = .09. There was no interaction between Association and Auditory stimulus intensity, *p* = .40, between Block type, Association and Auditory stimulus intensity, *p* = .42, between Stimulus type, Association, and Auditory stimulus intensity, *p* = .53, or between Block type, Stimulus type, Association, and Auditory stimulus intensity, *p* = .53. There were no other significant effects. These findings confirmed that there are no main nor interaction effects of auditory stimulus intensity on the SPE in the raw RTs.

## Appendix 6

In the present study, participants completed blocks of trials blocked by stimulus modality type prior to completing the blocks of trials with all the stimulus modality types intermixed. Familiarity with both the matching task and the 16 stimulus-response mappings (2 × Association × 2 Match-type × 4 stimulus types) gained during the blocked trials enabled participants to complete the blocks of intermixed trials which were otherwise too difficult. Notably, the increased familiarity of the stimuli and the S-R mappings gained in the blocked trials might be expected to increase the SPE in intermixed trials. However, additional analyses were conducted to test whether the magnitude of the SPE significantly decreased across individual blocks within each of the Blocked and Intermixed conditions of the Matching task. Given that Stimulus modality type was balanced across individual blocks, and our previous analyses indicated that Auditory stimulus intensity had no significant effect, the Stimulus modality type (V+VL, A+AL, A+VL, V+AL) and Auditory stimulus intensity (50 dB, 70dB) conditions were collapsed. Self-bias index scores in RT were then submitted to a one-way repeated-measures ANOVA (Block number: 1, 2, 3, 4) to compare the SPE across the individual blocks of trials blocked by stimulus modality type. There was one studentized residuals outlier (studentized residuals outside ∓ 3.0 in absolute value), and one condition was not normally distributed. With the outlier included (N = 48), the analysis revealed no significant main effect of Block, *F*(3, 141) = 2.40, *p* = .07, η_p_^2^ = .05, indicating that the magnitude of the SPE did not decrease significantly across blocks. With the outlier excluded from the analysis (N = 47), this finding was replicated, *F*(3, 138) = 2.38, *p* = .07, η_p_^2^ = .05.

Self-bias index scores in RT were then submitted to a one-way repeated-measures ANOVA (Block number: 1, 2, 3, 4, 5) to compare the SPE across the individual blocks of intermixed trials (N = 48). There was one studentized residuals outlier, and one condition was not normally distributed. With the outlier included (N = 48), the analysis revealed no significant main effect of Block, *F*(1.21, 56.94) = 0.54, *p* = .70, η_p_^2^ = .01 (Greenhouse-Geisser correction), indicating that the magnitude of the SPE did not decrease significantly across blocks. With the outlier excluded from the analysis (N = 47), all conditions were normally distributed, and this finding was replicated, *F*(3.33, 153.10) = 1.40, *p* = .24, η_p_^2^ = .03 (Greenhouse-Geisser correction).
