# Supplementary material for: Self-prioritization with unisensory and multisensory stimuli in a matching task
Source: Atten Percept Psychophys. 2022 May 10;84(5):1666–88. doi: 10.3758/s13414-022-02498-z (PMC9232425; doi:10.3758/s13414-022-02498-z)
Supplement: Supplementary file 1 — (PDF 294 kb) [file 13414_2022_2498_MOESM1_ESM.pdf]

Table A1

F-statistics for self-bias index scores in RT and *d′* assessed using 2 (between-groups Auditory stimulus intensity: 50 dB, 70 dB) x 2 (within-groups Block type: Blocked, Intermixed) x 4 (within-groups Stimulus type: V+VL, A+AL, A+VL, V+AL) mixed factorial ANOVA.

	**SPE RT**	**SPE *d′***
Block type	*$F(1, 48) = 15.51, p < .001, \eta p^2 = .24$	$F(1, 48) = 0.25, p = .62, \eta p^2 = .01$
Block type x Intensity	$F(1, 48) = 0.04, p = .85, \eta p^2 = .00$	$F(1, 48) = 3.68, p = .06, \eta p^2 = .07$
Stimulus type	*$F(2.32, 111.30) = 4.49, p = .01, \eta p^2 = .09$	$F(3, 144) = 0.17, p = .92, \eta p^2 = .00$
Stimulus type x Intensity	$F(3, 144) = 0.65, p = .59, \eta p^2 = .01$	$F(3, 144) = 0.58, p = .63, \eta p^2 = .01$
Block type x Stimulus type	$F(3, 144) = 2.46, p = .06, \eta p^2 = .05$	$F(3, 144) = 0.24, p = .87, \eta p^2 = .01$
Block type x Stimulus type x Intensity	$F(3, 144) = 0.58, p = .63, \eta p^2 = .01$	$F(3, 144) = 0.78, p = .51, \eta p^2 = .02$

Notes. N = 50. Outliers included (NB findings were replicated with outliers excluded—see main text). V+VL = Visual-shape+Visual-Label, A+AL = Auditory-object+Auditory-Label, A+VL = Auditory-object+Visual-Label , V+AL = Visual-shape+Auditory-Label. Intensity = Auditory stimulus intensity. *Significant F-statistics with $p < .05$.
