# Supplementary material for: Self-prioritization with unisensory and multisensory stimuli in a matching task
Source: Atten Percept Psychophys. 2022 May 10;84(5):1666–88. doi: 10.3758/s13414-022-02498-z (PMC9232425; doi:10.3758/s13414-022-02498-z)
Supplement: Supplementary file 2 — (PDF 316 kb) [file 13414_2022_2498_MOESM2_ESM.pdf]

Table A2

F-statistics for multisensory (percentage) gains/costs for RT and *d′* assessed using 2 (between-groups Auditory stimulus intensity: 50 dB, 70 dB) x 2 (within-groups Block type: Blocked, Intermixed) x 2 (within-groups AV Stimulus type: A+VL, V+AL) x 2 (within-groups Association: self, stranger) mixed factorial ANOVA. Pairwise comparisons for significant interactions also presented.

			MS percentage gains/costs RT	MS gains/costs *d′*
Block type			*$F(1, 48) = 9.59$, $p = .003$, $\eta p^2 = .17$	$F(1, 48) = 0.34$, $p = .57$, $\eta p^2 = .01$
Block type x Intensity			$F(1, 48) = 0.01$, $p = .94$, $\eta p^2 = .00$	$F(1, 48) = 0.54$, $p = .47$, $\eta p^2 = .01$
AV Stimulus type			*$F(1, 48) = 45.63$, $p < .001$, $\eta p^2 = .49$	*$F(1, 48) = 22.07$, $p < .001$, $\eta p^2 = .32$
AV Stimulus type x Intensity			$F(1, 48) = 0.95$, $p = .33$, $\eta p^2 = .02$	$F(1, 48) = 1.48$, $p = .23$, $\eta p^2 = .03$
Association			$F(1, 48) = 0.05$, $p = .83$, $\eta p^2 = .00$	$F(1, 48) = 0.06$, $p = .81$, $\eta p^2 = .00$
Association x Intensity			$F(1, 48) = 0.31$, $p = .58$, $\eta p^2 = .01$	$F(1, 48) = 0.03$, $p = .86$, $\eta p^2 = .00$
Block type x AV Stimulus type			$F(1, 48) = 0.92$, $p = .34$, $\eta p^2 = .02$	*$F(1, 48) = 6.56$, $p = .01$, $\eta p^2 = .12$
Block type x AV Stimulus type x Intensity			$F(1, 48) = 0.00$, $p = .95$, $\eta p^2 = .00$	$F(1, 48) = 0.41$, $p = .52$, $\eta p^2 = .01$
Block type x Association			*$F(1, 48) = 6.15$, $p = .02$, $\eta p^2 = .11$	$F(1, 48) = 0.02$, $p = .90$, $\eta p^2 = .00$
Block type x Association x Intensity			$F(1, 48) = 0.15$, $p = .70$, $\eta p^2 = .00$	$F(1, 48) = 0.10$, $p = .76$, $\eta p^2 = .00$
AV Stimulus type x Association			*$F(1, 48) = 4.56$, $p = .04$, $\eta p^2 = .09$	$F(1, 48) = 0.47$, $p = .50$, $\eta p^2 = .01$
AV Stimulus type x Association x Intensity			$F(1, 48) = 0.76$, $p = .39$, $\eta p^2 = .02$	$F(1, 48) = 0.04$, $p = .84$, $\eta p^2 = .01$
Block type x AV Stimulus type x Association			$F(1, 48) = 0.26$, $p = .61$, $\eta p^2 = .01$	$F(1, 48) = 0.02$, $p = .88$, $\eta p^2 = .00$
Block type x AV Stimulus type x Association x Intensity			$F(1, 48) = 0.07$, $p = .79$, $\eta p^2 = .00$	$F(1, 48) = 0.00$, $p = .97$, $\eta p^2 = .00$
**Pairwise comparisons**				
**Block types**	**Stimulus types**	**Associations**		
Blocked		Self & Stranger	$p = .08$	
Intermixed		Self & Stranger	$p = .06$	
Blocked & Intermixed		Self	**$p < .001$	
Blocked & Intermixed		Stranger	$p = .38$	
	A+VL & V+AL	Stranger	**$p = .001$	
	A+VL & V+AL	Self	**$p < .001$	
	A+VL	Self & Stranger	$p = .18$	
	V+AL	Self & Stranger	$p = .05$	
Blocked	A+VL & V+AL			**$p = .04$
Intermixed	A+VL & V+AL			**$p < .001$

Notes. N = 50. Outliers included (NB findings were replicated with outliers excluded—see main text). AV = audiovisual. V+VL = Visual-shape+Visual-Label, A+AL = Auditory-object+Auditory-Label, A+VL = Auditory-object+Visual-Label , V+AL = Visual-shape+Auditory-Label. Intensity = Auditory stimulus intensity. MS = multisensory. *Significant F-statistics with $p < .05$. **Significant p-values following a Holm-Bonferroni correction (unadjusted significance values reported).
